# Preclinical evaluation of AAV9-coSMN1 gene therapy for spinal muscular atrophy: efficacy and safety in mouse models and non-human primates

**DOI:** 10.1186/s10020-025-01207-4

**Published:** 2025-04-29

**Authors:** Wenhao Ma, Zhijie Wu, Tianyi Zhao, Yan Xia, Jing Qin, Xue Tian, Xin Li, Jun He, Yan Zhang, Lina Zhang, Li Li, Zheyue Dong, Zhichun Feng, Xiaoyan Dong, Wang Sheng, Xiaobing Wu

**Affiliations:** 1 Beijing GeneCradle Therapeutics Inc, Beijing, 100176 China; 2 Beijing Ruicy Institute of Gene Therapy for Rare Disease, Beijing, 102629 China; 3 Beijing University of Technology, Beijing, 100124 China; 4 Faculty of Pediatrics, the Chinese PLA General Hospital, Beijing, 100700 China; 5 National Engineering Laboratory for Birth Defects Prevention and Control of Key Technology, Beijing, 100700 China

## Abstract

**Background:**

Spinal muscular atrophy (SMA) is a severe neuromuscular disorder caused by the loss of motor neurons in the spinal cord. Our team has initiated clinical trials using adeno-associated virus serotype 9 (AAV9) vectors carrying a codon-optimized human SMN1 (coSMN1) gene, delivered via intrathecal (IT) injection. Here, we present the preclinical research that laid the groundwork for these trials, offering comprehensive data on the efficacy and safety of AAV9-coSMN1 in both murine models and non-human primates.

**Material and method:**

We developed a codon-optimized *hSMN1* expression cassette and analyzed SMN protein levels using Western blot and immunofluorescence. Taiwanese SMA-like mouse model was employed to assess tail length preservation, as well as to examine motor neuron and skeletal muscle pathological phenotypes through immunofluorescence and histopathological staining. Serum biomarkers in both mice and cynomolgus monkeys were measured using a blood chemistry analyzer. The in-vivo biodistribution of AAV9-coSMN1 and toxicological profile were investigated through quantitative Polymerase Chain Reaction(qPCR) and histopathological staining.

**Results:**

Codon optimization of *hSMN1* led to enhanced gene expression and increased SMN protein levels in vitro. AAV9-coSMN1 demonstrated significant therapeutic efficacy in a Type 3 SMA mouse model, effectively rescuing motor neurons, preserving tail integrity, and improving skeletal muscle histopathology. In vivo studies, both mice and cynomolgus monkeys revealed widespread CNS distribution following a single intracerebroventricular or intrathecal injection, with no observed toxic inflammatory responses in the dorsal root ganglia. Peripheral organs also showed detectable levels of the vector gene, indicating effective systemic distribution.

**Conclusion:**

The preclinical evaluation confirms that AAV9-coSMN1 is a safe and effective therapeutic candidate for SMA, with potential applicability across various phenotypes. The study provides critical data supporting its advancement to clinical trials, underscoring its promise for broader neurological applications.

**Supplementary Information:**

The online version contains supplementary material available at 10.1186/s10020-025-01207-4.

## Introduction

SMA is a genetic neuromuscular disorder characterized by the progressive degeneration of motor neurons in the spinal cord and brainstem, leading to muscle weakness and atrophy. The underlying cause of SMA is a mutation in the* SMN1* gene, which results in insufficient production of the SMN protein, essential for motor neuron health and function (Mercuri, E. et al. 2020).

Several therapeutic strategies have been developed to address this debilitating condition (Mercuri, E. et al. 2022). Risdiplam, an orally administered small molecule, modifies the splicing of *SMN2* pre-mRNA, leading to increased production of functional SMN protein (Paik, J. 2022). Nusinersen, an antisense oligonucleotide administered intrathecally, also enhances *SMN2* splicing to increase SMN protein levels (Hoy, S.M. 2021). While both therapies have shown clinical efficacy, challenges remain, including the invasive delivery method of Nusinersen and the need for continuous administration in both treatments (Oskoui et al. 2023; Mercuri E et al. 2018).

Zolgensma (onasemnogene abeparvovec) represents a significant advancement in SMA treatment as a gene therapy that delivers a functional copy of the *SMN1* gene using an AAV9 vector. Administered as a one-time intravenous infusion, Zolgensma has demonstrated substantial improvements in motor function and survival in SMA patients (Mendell J R et al. 2017) (https://www.fda.gov/vaccines-blood-biologics/zolgensma). However, the therapy has raised concerns regarding liver toxicity and immune responses, particularly in older patients (Chand D et al. 2021; Ruggiero R et al. 2024), which means it is only suitable for young children below a certain weight. Hence, a new intrathecal formulation, coded as OAV101 IT is being developed.

Given these developments, there is a need for further research into gene therapies that can offer broad and sustained efficacy across all SMA types with minimal side effects. GC101, an AAV9 vector-based gene therapy delivering a co*SMN1* gene, has entered clinical trials for SMA types 1 (Ma X et al. 2024), 2, and 3 (NCT05824169||2023.02.25; NCT05901987||2023.08.01; NCT06421831||2024.05.10), delivered via intrathecal (IT) injection. This study presents the preclinical evaluation of GC101, focusing on its efficacy in SMA mouse models and safety in both murine models and non-human primates, to support its continued clinical development.

## Materials and methods

### Plasmids construct

The human* SMN1* gene and codon optimized *SMN1* gene (OptimumGene^™^) were synthesized and cloned into pUC57 vector. The pITR-CA-coSMN1 and pITR-CA-hSMN1 plasmids were obtained by cloning promoter and gene (Supplemental appendix) into baculovirus shuttle plasmids containing wild type AAV2 packaging element ITR by restriction enzyme. The recombinant baculovirus plasmid (Bacmid) was obtained by transforming these two plasmids into DH10Bac strains.

### AAV vector production

AAVDJ serotype vectors are packaged using three-plasmid co-transfection system, briefly outlined as transient transfection of HEK293 cells using an adenovirus helper plasmid, a packaging plasmid (containing the gene sequence of AAVDJ shell and rep protein associated with AAV2 replication), and one of the pITR-CA-coSMN1 or pITR-CA-SMN1 plasmids. The cells were collected after about 72 h. The production of rAAV9-coSMN1 vector was achieved using the Bac-to-AAV production system. One auxiliary baculovirus contained the coSMN1 expression cassette, and the other carried the AAV9 capsid (cap9) and the sequence of the replication-associated protein gene (AAV2 rep). The vector was obtained by co-infection of Sf9 cells with two auxiliary baculoviruses. The cells were collected after about 72 h. AAV vector was purified by CsCl ultrafast centrifugation after cell lysis with cell harvest solution The purity of the AAV virus was assessed by SDS-PAGE, and the physical titer was determined using qPCR with specific probes.

### Animals

The SMA 3 model mice (*Smn1*^−/−^, *SMN2*^+/+^) used in this study were purchased from Jackson Laboratory (Stock No: 005058), is and a rodent SMA animal model, also named as known as an SMA-like mouse model (HM Hsieh-Li et al. 2000). The C57BL/6N mice used in this study were derived from Beijing Union-Genius Pharmaceutical Technology Co., Ltd.. Two and half years old nonhuman primates, Cynomolgus Monkey, were used in this study. Feeding and management followed the standard operating procedures (SOP) of Beijing JOINN New Drug Research Center Co., Ltd. All operations and activities involving the use and welfare of animals were approved by the Institutional Animal Care and Use Committee (IACUC) of corresponding companies prior to operation. Neonatal mice (PND1) were anesthetized on ice and administered AAV9-coSMN1 by unilateral injection with a Hamilton syringe into the lateral ventricle (33 G). Intracerebroventricular coordinates for PND1 mice: X = 1.0 mm lateral to bregma, Y = 0.3 mm posterior, Z = 2.0 mm depth (adjusted for neonatal skull thickness). The total volume of injection was 5 μL/pup. After the injection, the mice were rewarmed until they returned to normal temperature and could move their limbs autonomously, autonomously and then was returned to the cage with its mother (Kim J Y et al. 2014). The nonhuman primates with a AAV9 neutralizing Ab of ≤ 1:50 were confirmed before treatment and anesthetized using intramuscular injection of ketamine at a dose of 7.5 mg/kg, and the animals were fasted for at least 6 h before anesthesia. After anesthesia, a 1 mL disposable sterile syringe and matching needle was used to intrathecally inject the appropriate volume of AAV9-coSMN1 or saline. The total volume of injection was 800 μL/animal.

### Western blot analysis

Cultured BHK21 or HEK293 cells in a six-wells plate with DMEM medium (11995040, Thermo, USA) containing 10% serum (SERD76294-180, VWR Seradigm, USA) until 80% confluent, then Utilized lipofectamine 2000 (11668030,Thermo,USA) to transfect with 4 μg of the pITR-CA-coSMN1 or pITR-CA-SMN1 plasmids and harvest the cells 48 h later. Cells were collected (tissues were collected after grinding with a cryogenic freezer grinder) into a centrifuge tube, total proteins were extracted by adding RIPA lysis buffer (R0020, Solarbio, China) containing a protease inhibitor (PMSF, P0100, Solarbio, China), and protein concentrations were quantified by the BCA method (23225, Thermo, USA). Equal amounts of protein separated by 12.5% SDS-PAGE gel, then transferred to a PVDF membrane by a semidry transmembrane apparatus. The membranes were blocked with TBST containing 5% skim milk powder and then incubated with mouse anti-SMN antibody (1:1000 dilution, 610646, BD, USA) or mouse anti-βtubulin antibody (1:10000 dilution, TA-10, ZSGB-BIO, China) at 37 °C for 1 h. Washed gently with TBST buffer and incubated with HRP conjugated goat anti-mouse IgG antibody (1:5000 dilution, GB-2305, ZSGB-BIO, China) at 37 °C for 1 h. Highly sensitive ECL luminescent reagent (C500044-0100, Sangon Biotech, China) was used for imaging on Tanon 4800 automatic chemiluminescence image analysis system.

### Immunostaining analysis

U87-MG or NB1 cells were cultured on tissue culture (TC)-treated glass slides in 6-well plate. Cell growth reached 30% confluent, infected with 5000 MOI by AAVDJ-CA-coSMN1 or AAVDJ-CA-SMN1. Incubated 48 h, the supernatant was removed and the cells were washed with PBS, and the cell slides were fixed with 4% paraformaldehyde (PFA).

For the collected tissue, which were placed in 4% PFA in a refrigerator at 2–8 °C for 24 h. Then, the tissues were removed and dehydrated in 30% sucrose for at least 48 h. The tissue was embedded in optimal cutting temperature compound (OTC) and sectioned into 5 μm thick frozen sections.

Cell and tissue sections were permeated with 0.1% Triton X-100 (T8200, Solarbio, China) in PBS. Antigen retrieval was then performed in citrate buffer (10 mM citric acid, pH 6.0) at 95 °C for 30 min. After cooling and washing in PBS, sections were incubated with blocking reagent for 1 h. Primary antibodies (Anti-SMN, 1:200 dilution, 610646, BD, USA; Anti-choline acetyltransferase (ChAT), 1:200 dilution, ab178850, Abcam, UK) added to the sections, and incubated overnight at 4 °C. The sections were washed 3 times and then incubated with the secondary antibody with appropriate signal marker coupling. For immunofluorescence staining using fluorescently labeled secondary antibodies, (Dylight 549 Goat Anti-Mouse IgG, A23310, DyLight 488 Goat Anti-Mouse IgG, A23210, 1:200 dilution, Abbkine, USA), and the nuclei were labeled with DAPI, and the results were recorded by fluorescence microscopy. Immunohistochemical staining was performed with secondary antibodies labeled with HRP (1:5000 dilution, ZB-2305, ZSGB-BIO, China). After DAB color development, the nucleus was labeled with hematoxylin, and the experimental results were photographed with a common optical microscope.

The Image J software was used to analyze the immunostaining photos. For cell samples of each infection condition, we set up 3 independent repeat holes to prepare cell slides. Each climbing film sample was randomly selected in 3 different fields for image acquisition during microscope observation, and 3 typical cells in each field were selected for quantitative analysis of fluorescence intensity. Through this stratified sampling method (3 dots × 3 field of view × 3 cells), a total of 27 independent regions of cell fluorescence intensity data were obtained for each experimental condition. ChAT immunofluorescence of the spinal cord of SMA3 mice was carried out by analyzing the number of positive cells in 3 photos of different mice. Immunohistochemical images of C57BL/6N mice were performed by analyzing the positive signal intensities of the entire image.

### Histopathological staining

Tissues from animals were fixed with Davidson's solution or 10% neutral formalin, embedded in paraffin, sectioned, and stained with hematoxylin–eosin. All tissue sections were examined, diagnosed, and classified, with lesions graded on a 5-level scale: minimal, mild, moderate, severe, and very severe. ATPase (pH 4.5) and succinate dehydrogenase (SDH) staining utilized frozen section technique, the muscle tissue sections of SMA and control mice quadriceps and gluteus maximus were followed by staining kit (G2380 and G2000, Solarbio, China) operation protocol. For ATPase (pH 4.5) staining, Type I muscle fibers are stained dark under acidic conditions. The area of muscle fibers was analyzed by the ImageJ software. The SDH staining signal strength of muscle fibers was analyzed using ImageJ software, and three photos from different regions were analyzed for each group of animals.

### Serum biochemistry assay

For the serum biochemistry assay, blood was collected from mice via the orbital venous plexus on D15, D43, and D92 after overnight fasting, collected 0.5 ml samples in tubes without anticoagulant, and analyzed with a BECKMAN COULTER AU480 automatic biochemical analyzer. Similarly, blood samples from the subcutaneous veins in the hindlimbs of non-human primates were collected on D-1 (before administration), D8, D15, D29, D57, and D91, also after overnight fasting, collected 1 ~ 2 ml serum samples in tubes without anticoagulant, and analyzed with a TBA-120FR automatic biochemical analyzer.

### Vector genome distribution

Tissue samples to be tested are collected and genomic DNA is extracted using the Genomic DNA Extraction Kit (DP304-03, TIANGEN, China). The concentration of gDNA was calculated by measuring OD value of the sample with microplate reader, and the distribution of carrier genome in blood and tissue was detected by fluorescence quantitative PCR.

### Statistical analysis

Data are presented as the mean ± standard deviation. All the data obtained in the experiment were input into Excel for calculation and analyzed using GraphPad prism. The analysis steps were as follows: (1) Kolmogorov–Smirnov method should be used for normality test, Levene median method for homogeneity of variance test, and One-Way ANOVA should be conducted. If the normality and homogeneity of variance test fail, then a non-parametric Kruskal–Wallis test is required. (2) If the ANOVA test results were significant (p ≤ 0.05), Dunnett t test was further used for multiple comparison test; If the results of ANOVA were not significant (p > 0.05), the statistics were over. (3) If the Kruskal–Wallis test results were significant (p ≤ 0.05), the Mann–Whitney test method was further used to conduct multiple comparison tests; If the result of Kruskal–Wallis test was not significant (p > 0.05), the statistics were over. Significant differences are indicated by *(*p < 0.05, **p < 0.01, ***p < 0.001, ****p < 0.0001).

## Results

### Codon optimization in the design of GC101 significantly enhances SMN protein expression across multiple cell lines

**Fig. 1:**
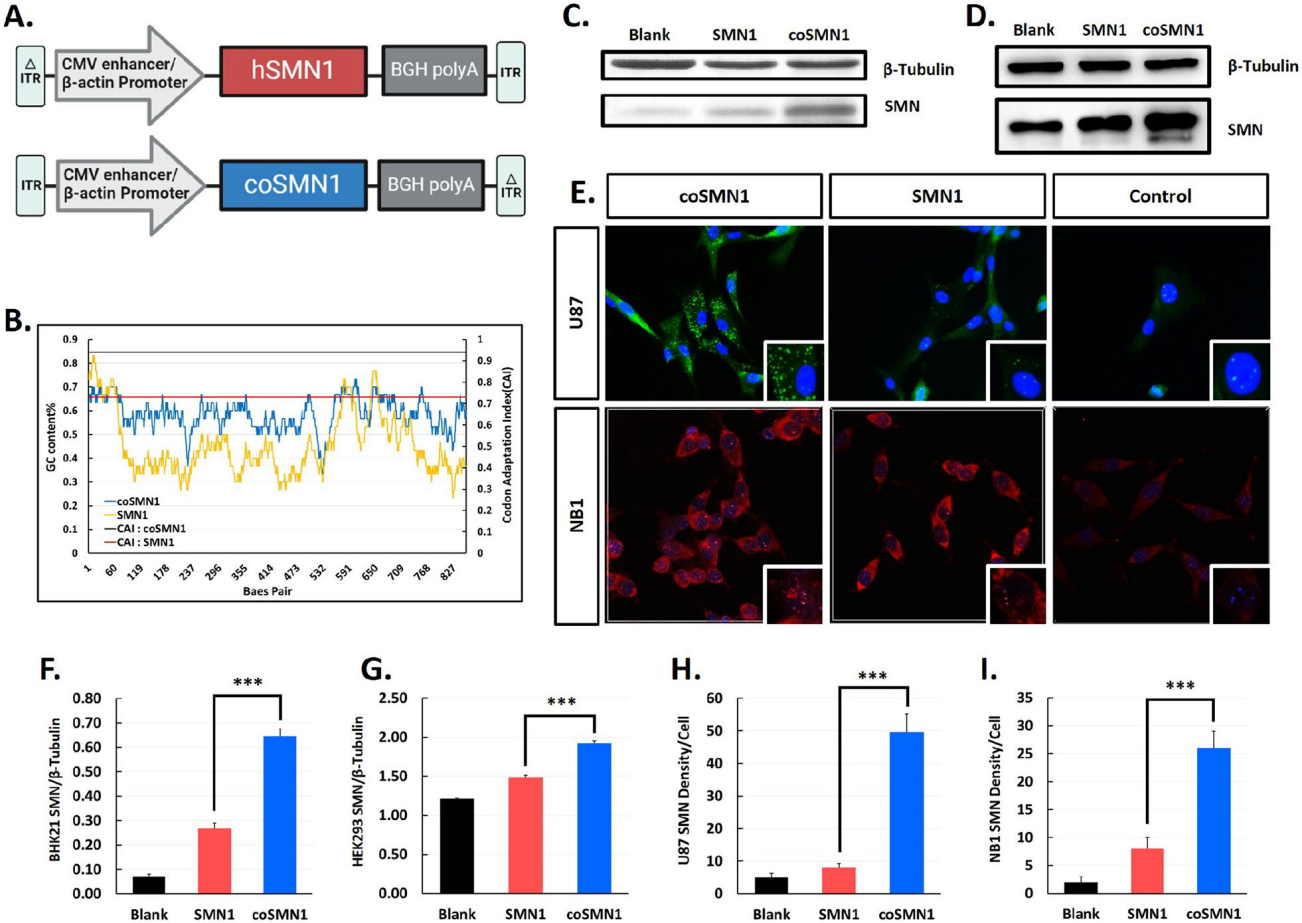
Codon-optimization of SMN reveals significant expression improved. **A** Schematics of CA-hSMN1 and CA-coSMN1 expression cassette. **B** Comparison of GC content and CAI in wild type *SMN1* cDNA and optimized sequence. The coSMN1 with more stable GC% content, and higher CAI score. GC content: SMN1 (yellow), coSMN1 (blue); CAI Index: SMN1 (Red line), coSMN1 (black line). **C-D** The expression of SMN protein in BHK-21 and HEK-293 cell lines was detected by western blotting. **E** The SMN protein level intensity in U87-MG cells and NB1 cells was analyzed by immunofluorescence. Blue: DAPI, Green: SMN, Red: SMN. **F**, **G** Quantitative analysis of western blot results of C and D, the SMN were normalized with β-Tubulin. H-I Quantitative analysis of immunofluorescence determine the mean intensity of SMN protein immunofluorescence in U87 and NB1 cells. Statistical analysis: Mean ± SD. **P < 0.01, ***P < 0.001. n = 3. Scale bars: 100 µm

Previous research has indicated that endogenous SMN protein levels are generally higher in the central nervous system (CNS) compared to peripheral tissues, with particularly elevated levels during early development (Wishart T M et al. 2010). Given the critical role of SMN protein in motor neuron survival, enhancing its expression in the CNS is essential for therapeutic effectiveness. The SMN protein, composed of 294 amino acids, includes a proline-rich domain that may contribute to DNA and mRNA secondary structure instability due to repetitive codon sequences (Fig. 1A).

To enhance the therapeutic potential of GC101, we optimized the codon adaptation index (CAI) of the SMN1 coding sequence, increasing it from 0.73 to 0.94, and improved the GC content from 45.20% ± 0.13 to 58.52% ± 0.07 (Fig. 1B) by utilizing the human codon preference database (Supplemental appendix). We then generated the plasmids pITR-CA-coSMN1 and pITR-CA-SMN1 for subsequent vector production and testing. The plasmids were transiently transfected into baby hamster kidney (BHK21) cells and human embryonic kidney (HEK293) cells to compare SMN protein expression levels. As expected, the pITR-CA-coSMN1 plasmid led to a significant increase in SMN protein expression in both cell lines (Fig. 1C, D). Specifically, in HEK293 cells, coSMN1 expression was 2.4-fold higher compared to natural SMN1, while in BHK21 cells, the increase was 1.3-fold (Fig. 1F, G).

To further validate the enhanced expression of coSMN1 in human neuronal cells, we opted to use the AAVDJ serotype, which offers superior transduction efficiency in vitro compared to AAV9. We generated AAVDJ-CA-coSMN1 and AAVDJ-CA-SMN1 vectors using the HEK293-based triple plasmid transfection system. These vectors were then used to infect human U87-MG and NB1 cells at a multiplicity of infection (MOI) of 5000 (Fig. 1E). Immunofluorescence analysis demonstrated that cells infected with AAVDJ-CA-coSMN1 exhibited approximately six-fold higher SMN protein levels compared to those infected with the natural SMN1 sequence (Fig. 1H). In NB1 cells, coSMN1 expression resulted in a three-fold increase in SMN protein levels relative to the native SMN1 codons (Fig. 1I).

### Dose-Dependent rescue of phenotype in SMA Type 3 mouse model following ICV injection of AAV9-coSMN1

**Fig. 2:**
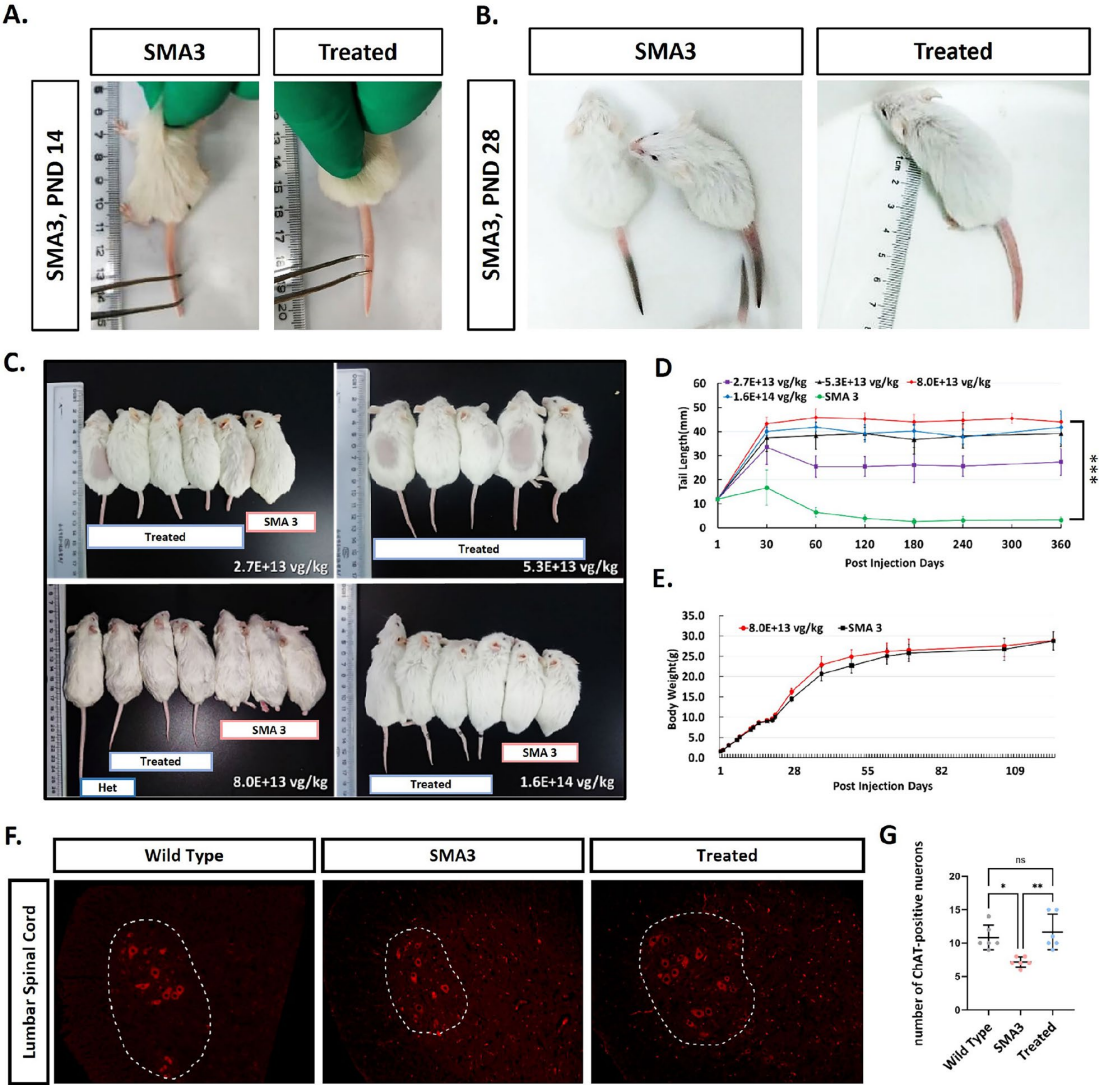
Motor neuron rescued and tail retention following AAV9-coSMN1 treatment one year. **A**, **B** The effects of AAV9-coSMN1 treatment on motor function and tail retention in SMA 3 model mice one year post-injection. SMA 3 mice display disease-related changes of tail morphology, highlighting improved tail length in treated mice. **C**, **D** The group comparisons of mice treated with various doses of AAV9-coSMN1 (2.7E+13, 5.3E+13, 8.0E+13, and 1.6E+14 vg/kg), demonstrating improved phenotype compared to SMA 3 controls. **E** The body weight change curve did not show any statistically significant differences. **F**, **G** Numbers of ChAT-positive motor neurons in treated mice at a dose of 5.3E+13 vg/kg compared to untreated controls and WT, confirming motor neuron rescued, n = 6. Statistical analysis: Mean ± SD. **P < 0.01, ***P < 0.001. Scale bars: 50 µm

To further validate the efficacy of AAV9-coSMN1, we treated SMA type 3 (SMA3) model mice with four different doses, ranging from 2.7E+13 to 1.6E+14 vg/kg, administered via intracerebroventricular (ICV) injection on postnatal day 1 (PND1). The untreated SMA3 model mice exhibited tail tip necrosis and gradual tail loss within 30 days after birth (Fig. 2A, B).

Notably, the treated mice displayed a dose-dependent preservation of tail length. At the lowest dose of 2.7E+ 13 vg/kg, the therapeutic effect was insufficient, with mice still showing partial tail necrosis. However, a significant improvement in tail length preservation was observed at a dose of 5.3E+13 vg/kg, and this effect was further enhanced at 8.0E+13 vg/kg (Fig. 2C, D). Interestingly, increasing the dose to 1.6E+14 vg/kg did not result in additional therapeutic benefit compared to the 8.0E+13 vg/kg group. Body weight analysis revealed no significant differences between the mice treated with 8.0E + 13 vg/kg and their untreated SMA3 littermate controls (Fig. 2E). Histological analysis of the lumbar spinal cord showed a significant preservation of motor neurons in the anterior horn region at a dose of 5.8E+13 vg/kg, as evidenced by ChAT staining (Fig. 2F, G).

These findings suggest that while AAV9-coSMN1 effectively rescues the SMA3 phenotype, there is an optimal dose range beyond which no further efficacy is achieved.

### AAV9-coSMN1 treatment improves skeletal muscle histopathology and mitochondrial function in SMA type 3 mice

In contrast to Type 1 SMA, where muscle atrophy progresses rapidly, Type 3 SMA exhibits a slower degeneration process. The myopathological features of Type 3 SMA include scattered small angular fibers and grouped atrophy, reflecting neurogenic changes. However, large-scale grouped atrophy, which is characteristic of Type 1 SMA, is significantly limited in Type 3 SMA. In the SMA Type 3 mouse model, similar pathological changes are observed, including variability in muscle fiber size and increased connective tissue (Kingma DW et al. 1991; Zalneraitis EL. 1991; Gendron N H et al. 1999).

**Fig. 3:**
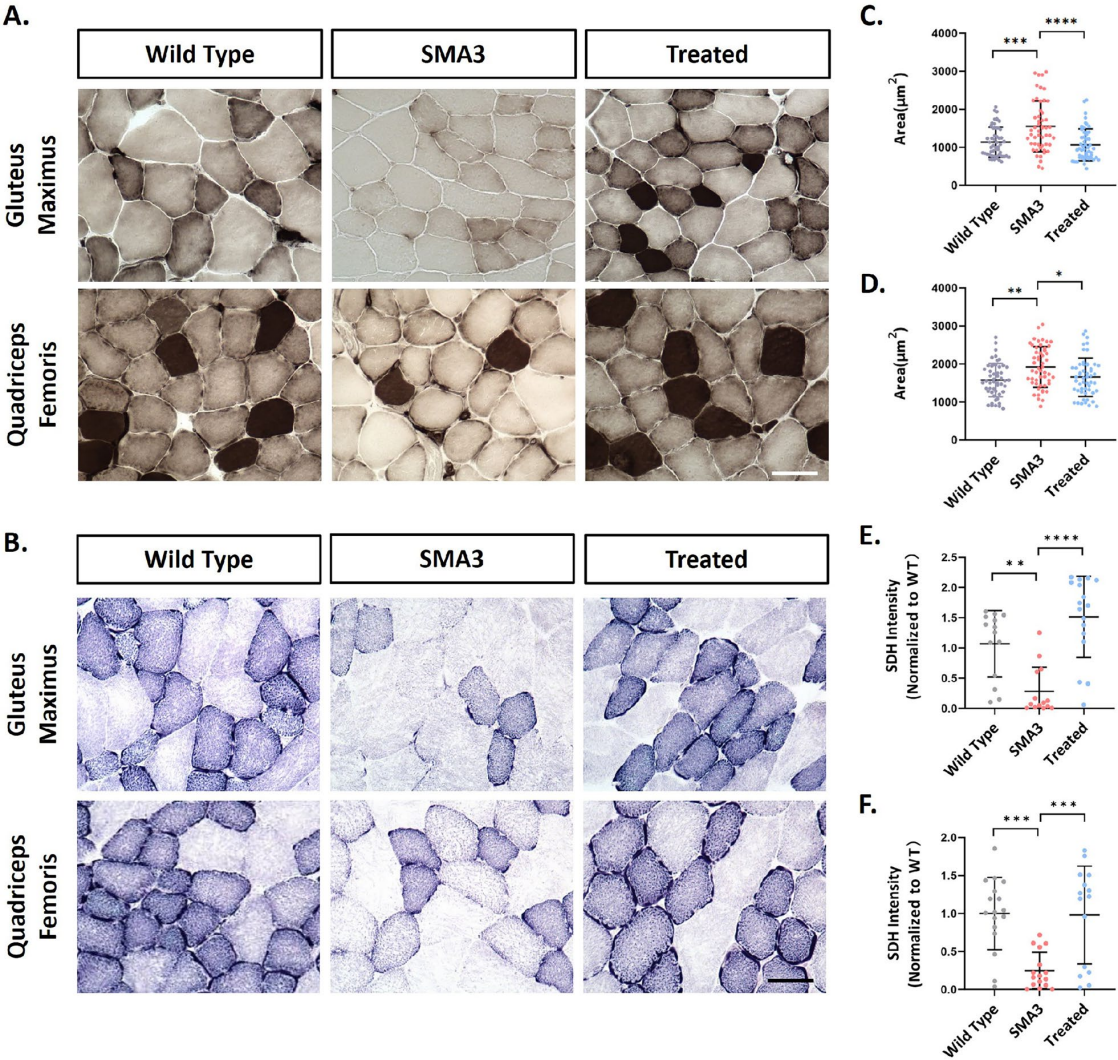
Skeletal Muscle Phenotype and Mitochondrial Function was improved in SMA3 Mice Following AAV9-coSMN1 treatment a dose of 5.3E+13 vg/kg at one year after treatment. **A** ATPase (pH 4.5) staining of gluteus maximus and quadriceps femoris muscle. **B** SDH staining of gluteus maximus and quadriceps femoris muscle. **C**, **D** Type I muscle fiber cross-sectional area analysis of gluteus maximus and quadriceps femoris muscle. **E**, **F** SDH staining intensity analysis of gluteus maximus and quadriceps femoris muscle, n=15. Statistical analysis: Mean ± SD. *P < 0.05, **P < 0.01, ***P < 0.001, ****P < 0.0001

To assess the therapeutic effects of AAV9-coSMN1 on skeletal muscle in this model, we analyzed muscle pathology in animals treated with a dose of 5.3E + 13 vg/kg. Our analysis revealed that, compared to wild-type controls, the overall ATPase (pH 4.5) staining intensity was reduced in the quadriceps femoris and gluteus maximus muscles of Type 3 SMA mice, indicating potential mitochondrial dysfunction (Fig. 3A). Meanwhile, the average muscle fiber area of intensely stained fibers was significantly increased. Similarly, the average area of type I or type II muscle fibers also showed a significant increase, suggesting compensatory hypertrophy in the skeletal muscle of type 3 SMA mice (Fig. 3C, D).

The timing for AAV9-coSMN1 administered at postnatal day 1, which is critical for SMA given the rapid progression of motor neuron loss. Furthermore, the average muscle fiber area was significantly restored to the levels comparable to those of wild-type controls, indicating a positive therapeutic effect on muscle function (Fig. 3C, D). Additionally, we observed a significant improvement in SDH activity (Fig. 3E, F), further supporting the restoration of mitochondrial function after treatment with AAV9-coSMN1.

These findings suggest that AAV9-coSMN1 not only ameliorates muscle atrophy in SMA Type 3 mice but also addresses underlying mitochondrial dysfunction, thereby improving overall muscle histopathology. The therapeutic impact of AAV9-coSMN1 when administered at later stages, particularly in less severe SMA patients need further study.

### ICV injection of AAV9-coSMN1 in C57BL/6N mice demonstrates dose-dependent safety and biodistribution

**Fig. 4:**
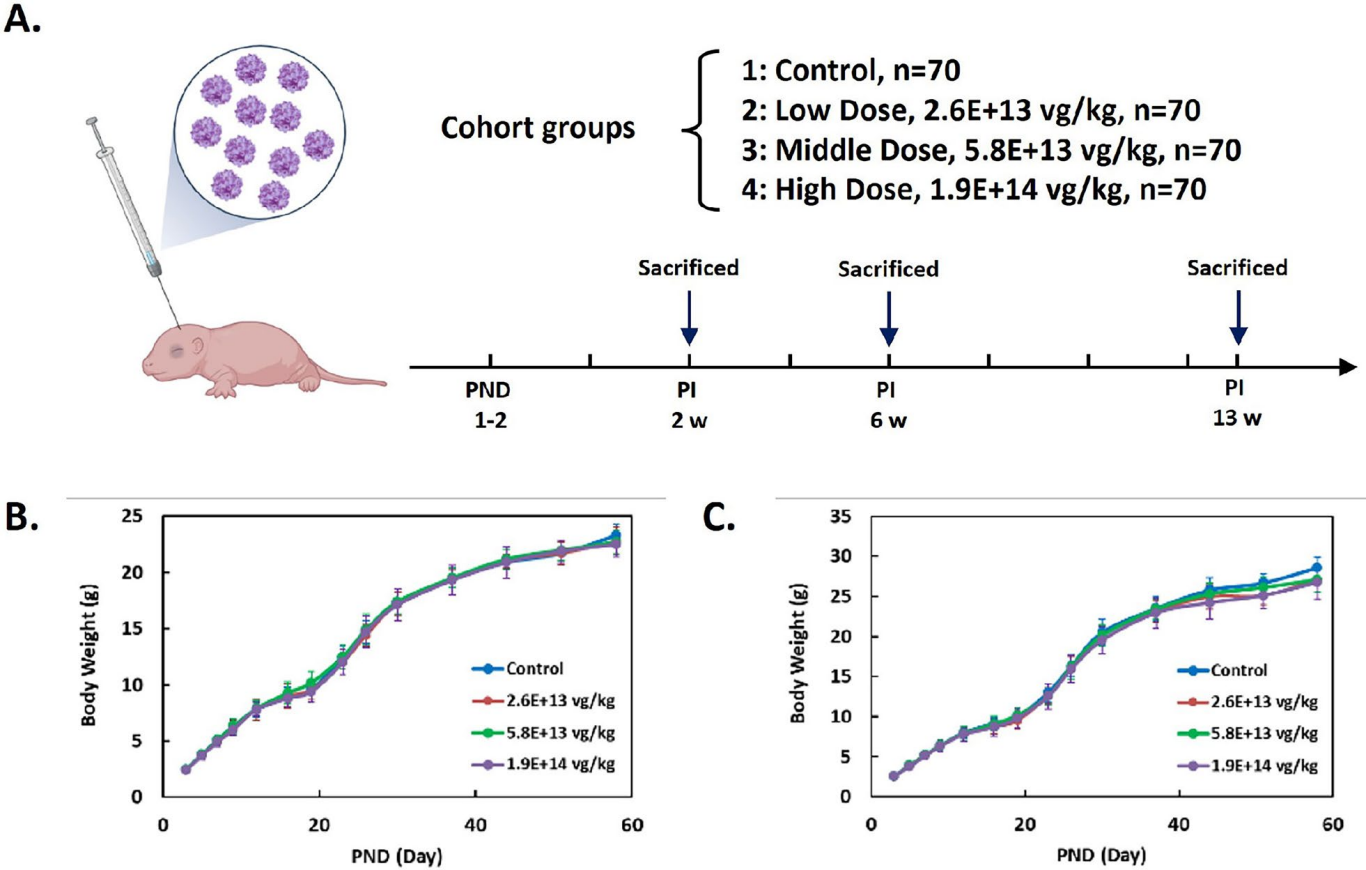
Cohort setup and body weight curves of the AAV9-coSMN1 toxicity study in C57BL/6N mice. **A** Schematic representation of the overall study design. AAV9-coSMN1 at various doses (2.6E+13 vg/kg, 5.8E+13 vg/kg, and 1.9E+14 vg/kg) via intracerebroventricular (ICV) injection in neonatal mice. Following the injection, mice were monitored over period, with specific time points for toxicity assessment. **B**, **C** The body weight curves of both male (left) and female (right) mice are shown. Statistical analysis: Mean ± SD

To further evaluate the safety and biodistribution profile of AAV9-coSMN1 in mice, we conducted a single-dose toxicity study under Good Laboratory Practice (GLP) conditions. The study included one control group and three treatment groups, with dosing levels of 2.6E+13 vg/kg, 5.8E+13 vg/kg, and 1.9E+14 vg/kg, respectively. Each group consisted of 70 animals (Fig. 4A). Newborn C57BL/6N mice received ICV injection of AAV9-coSMN1 and were monitored for body weight changes over 60 days.

The results showed no significant differences in body weight between the treated groups and the control group (Fig. 4B, C), indicating that the treatment was well tolerated across all dosage levels. Additionally, cage-side observations over 13 weeks demonstrated normal activity in all groups, with no significant variations in serum biochemical parameters, including ALT and AST levels, across different dosage groups (Tables 1, 2). Not only the CNS but also a significant systemic AAV biodistribution including liver, heart, lung, spleen, germinal epithelium, gastrointestinal epithelium and blood were observed (Supplementary Table 1). Additionally, a relative lower level of systemic biodistribution was indeed anticipated via CSF-mediated AAV infusion.

**Table 1:**
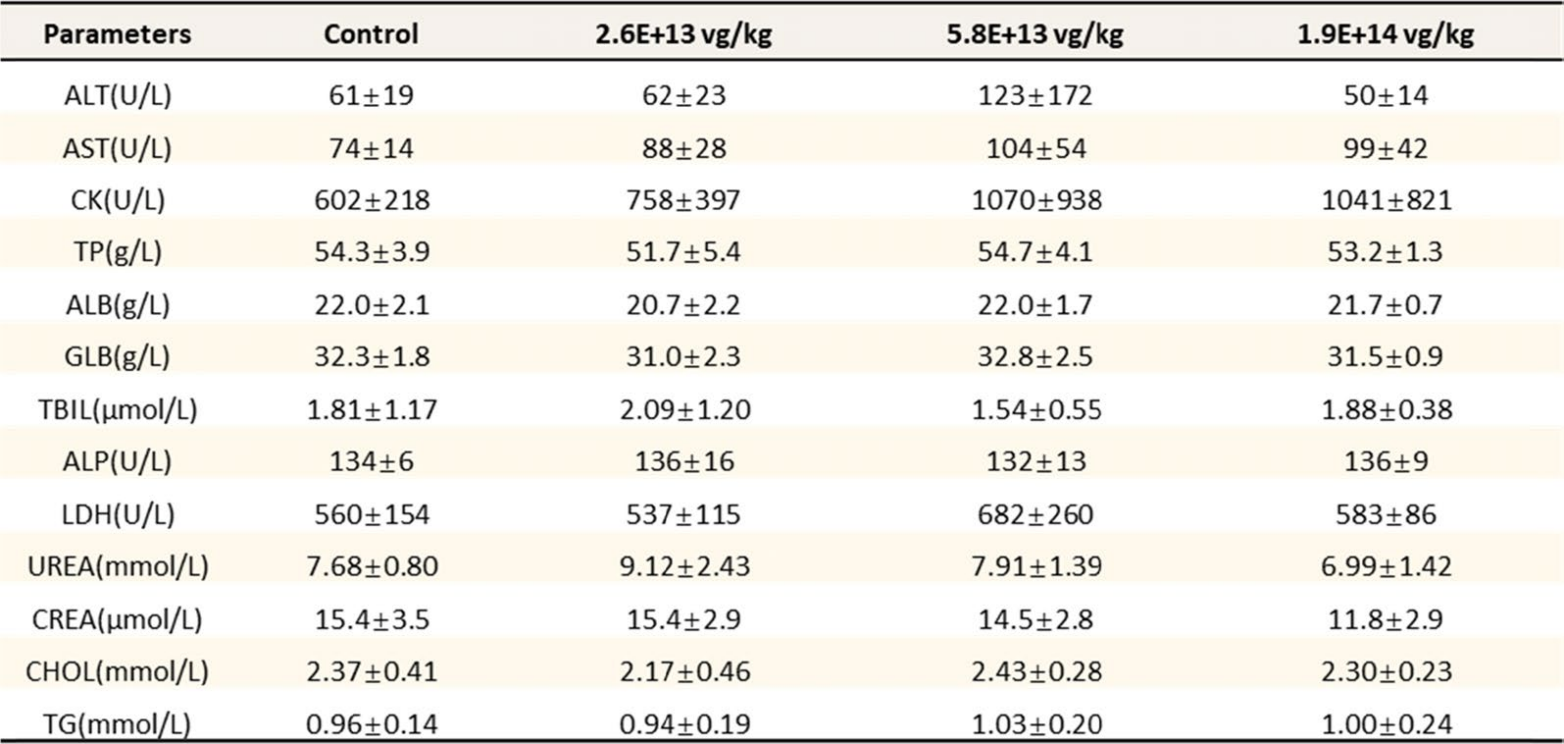
Serum biochemical parameters post injection 13 weeks in female mice

**Table 2:**
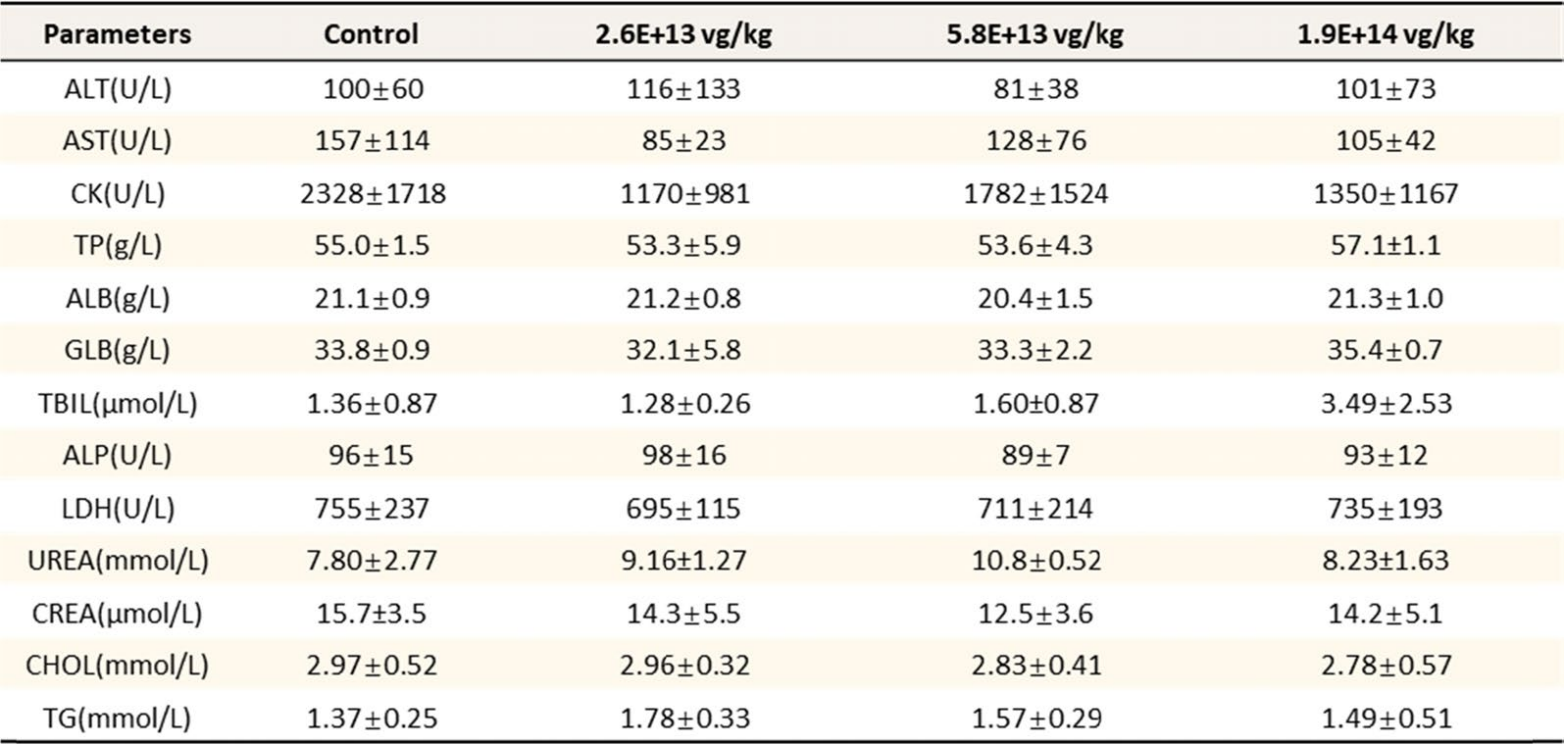
Serum biochemical parameters post injection 13 weeks in male mice

To further evaluate the biodistribution of AAV9-coSMN1 within the central nervous system of mice, we conducted an immunohistochemical analysis to assess SMN protein expression in the brain and spinal cord following ICV injection. The study included a control group and two treatment groups, receiving doses of 5.8E+13 vg/kg and 1.9E+14 vg/kg, respectively.

**Fig. 5:**
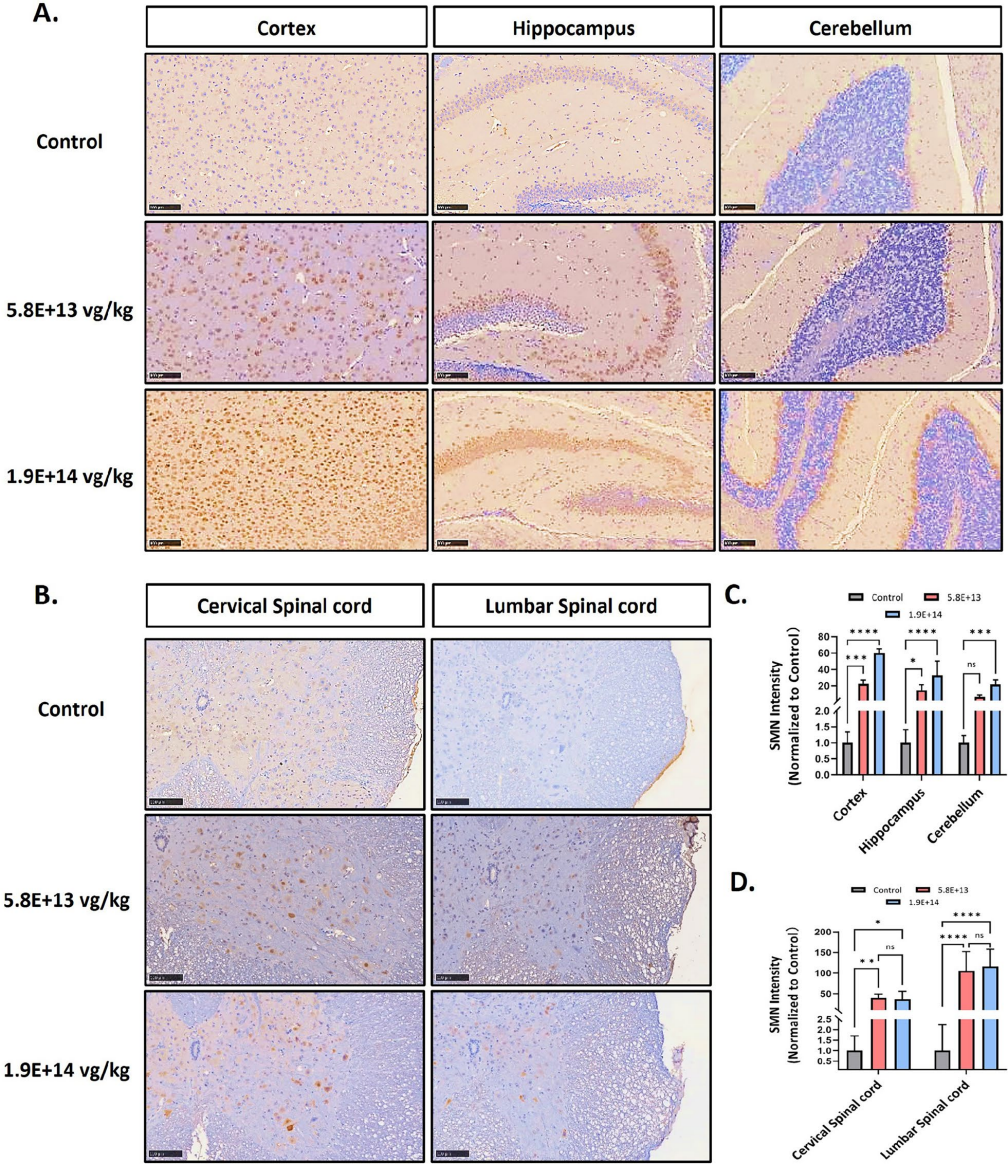
Biodistribution of SMN expression in the CNS following AAV9-coSMN1 ICV injection in mice on Day 60 posttreatment. **A**, **B** Immunohistochemical staining shows SMN expression in the cortex, hippocampus, cerebellum, cervical spinal cord, and lumbar spinal cord of mice treated with AAV9-coSMN1 at doses of 5.8E+13 vg/kg and 1.9E+14 vg/kg compared to controls. **C**, **D** Quantitative analysis reveals a dose-dependent increase in SMN expression across these central nervous system (CNS) regions, while no significant change is observed in the spinal cord. Statistical analysis: Mean ± SD. Significance is noted as *P < 0.05, **P < 0.01, ***P < 0.001, ****P＜0.0001， ns: not significant. Scale bars: 100 µm

The results demonstrated widespread SMN protein expression throughout the cortex, hippocampus, and cerebellum in treated mice, with signal intensity showing a dose-dependent increase (Fig. 5A, C). Similarly, strong SMN expression was observed in the cervical and lumbar regions of the spinal cord (Fig. 5B, D). However, further increasing the dosage from 5.8E+13 vg/kg to 1.9E+14 vg/kg did not result in a proportional increase in SMN expression, suggesting that a saturation point may have been reached at the lower dose.

These findings indicate that AAV9-coSMN1 achieves efficient and widespread SMN expression in the CNS, with a clear dose-dependent pattern, although higher doses may not confer additional benefit in terms of protein expression levels.

### Safety and biodistribution of AAV9-coSMN1 following intrathecal administration in cynomolgus monkeys

**Fig. 6:**
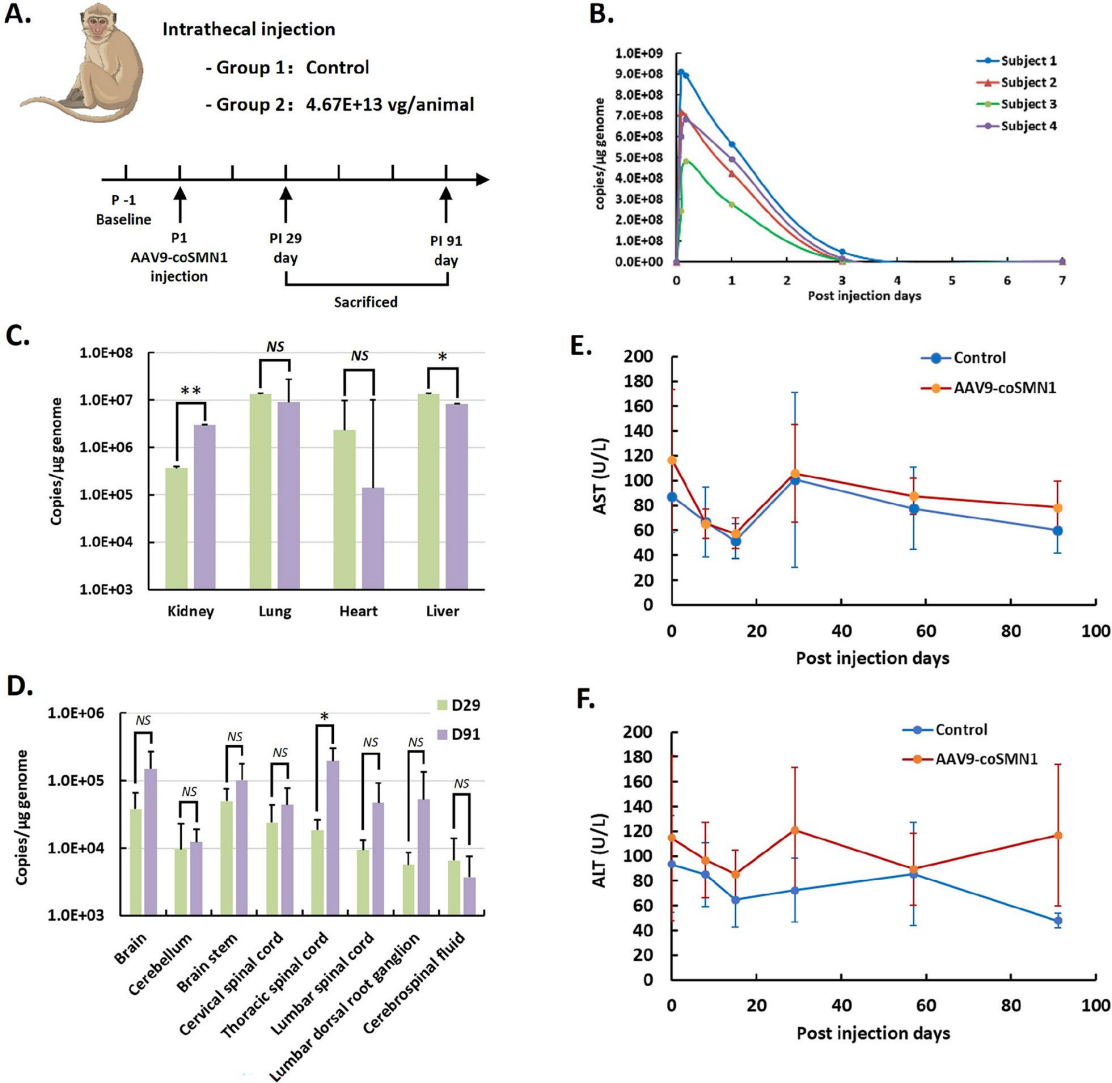
Biodistribution and Safety Profile of AAV9-coSMN1 in Non-Human Primates Following IT Injection. **A** Schematic of study design: AAV9-coSMN1 was administered via intrathecal (IT) injection in non-human primates with single dose. **B** Vector genome copy numbers in the plasma show a rapid decline within 7 days post-injection. **C** Biodistribution analysis in peripheral organs (kidney, lung, heart, and liver) on Day 29 and Day 91, demonstrating significant vector presence in the liver and kidney, with a notable reduction by Day 91. **D** Vector genome copy numbers in CNS tissues on Days 29 and 91 post-injection, showing sustained presence in the brain, spinal cord, and other neural tissues. **E**, **F** Serum AST (top) and ALT (bottom) levels indicate no significant liver toxicity compared to controls, suggesting a favorable safety profile. Statistical analysis: Mean ± SD. Significance noted as *P < 0.05, **P < 0.01, and ns: not significant

To evaluate the clinical safety and biodistribution of AAV9-coSMN1, we conducted a GLP compliant study involving a single intrathecal injection in 2.5-year-old cynomolgus monkeys (Fig. 6A). With reference to the animal studies of ZOLGENSMA, the dosage for intrathecal injection in NHP is 1E+13 vg/kg (Meyer K., 2015). Furthermore, toxicity in dorsal root ganglia (DRG) could observed in a dose range of 3E+12 to 1E+13 GC via intrathecal or intra-cisterna magna, or 1E+13 to 2E+14 GC/kg via intravenous injection as reported (Hordeaux J et al. 2024). Herein, each animal received a dose of 4.67E+13 vg/animal (1.87E+13 vg/kg each animal). Pharmacokinetic analysis revealed a rapid increase in vector genome concentration in the bloodstream shortly after administration, with peak levels observed within 10 min (Fig. 6B). By day four, concentrations returned to baseline and remained stable thereafter (Fig. 6B).

On days 29 and 91 post-injection, animals were euthanized for tissue collection. Vector genomes were detected in multiple peripheral organs, including the kidneys, lungs, heart, and liver, with the highest concentrations observed in the liver (Fig. 6C). Within the central nervous system, AAV9-coSMN1 showed widespread distribution, with vector genome copies exceeding 1E + 4 copies/μg DNA in the brain, cerebellum, spinal cord, and dorsal root ganglia (Fig. 6D), without significant temporal fluctuations.

Liver toxicity, particularly elevated transaminase levels, is a common concern with AAV-based gene therapies (Ruggiero R et al. 2024). However, in this study, no significant increases in AST or ALT levels were observed throughout the study period in the AAV9-coSMN1-treated group (Fig. 6E and F), suggesting a favorable hepatic safety profile. The immune responses against the AAV capsid or the transgene product, e.g., the anti-drug antibodies (ADA) were triggered two weeks after treatment (Supplementary Table 2) and persisted at a stable level, indicating a lower system exposure.

Considering the potential risk of dorsal root ganglia (DRG) inflammation following intrathecal AAV administration, we performed histopathological analysis of DRG tissue on day 91 post-injection. Hematoxylin and eosin (H&E) staining revealed no signs of inflammatory cell infiltration or neuronal necrosis in the treated monkeys. Immunohistochemical analysis further confirmed successful SMN protein expression in the DRG without any associated inflammation, indicating that AAV9-coSMN1 is both effective and safe in targeting CNS tissues.

These findings suggest that AAV9-coSMN1 has a favorable safety and biodistribution profile when administered intrathecally in cynomolgus monkeys, with no observed toxicity or adverse inflammatory responses.

### Histological safety evaluation of dorsal root ganglia and spinal cord following intrathecal administration of AAV9-coSMN1 in cynomolgus monkeys

To further assess the safety profile of AAV9-coSMN1, we conducted a histopathological analysis of the dorsal root ganglia (DRG) and spinal cord in cynomolgus monkeys following a single intrathecal administration of AAV9-coSMN1. Each monkey received a dose of 4.67E+13 vg/animal (approximately 1.87E+13 vg/kg), and tissues were collected on day 91 post-injection for analysis.

**Fig. 7:**
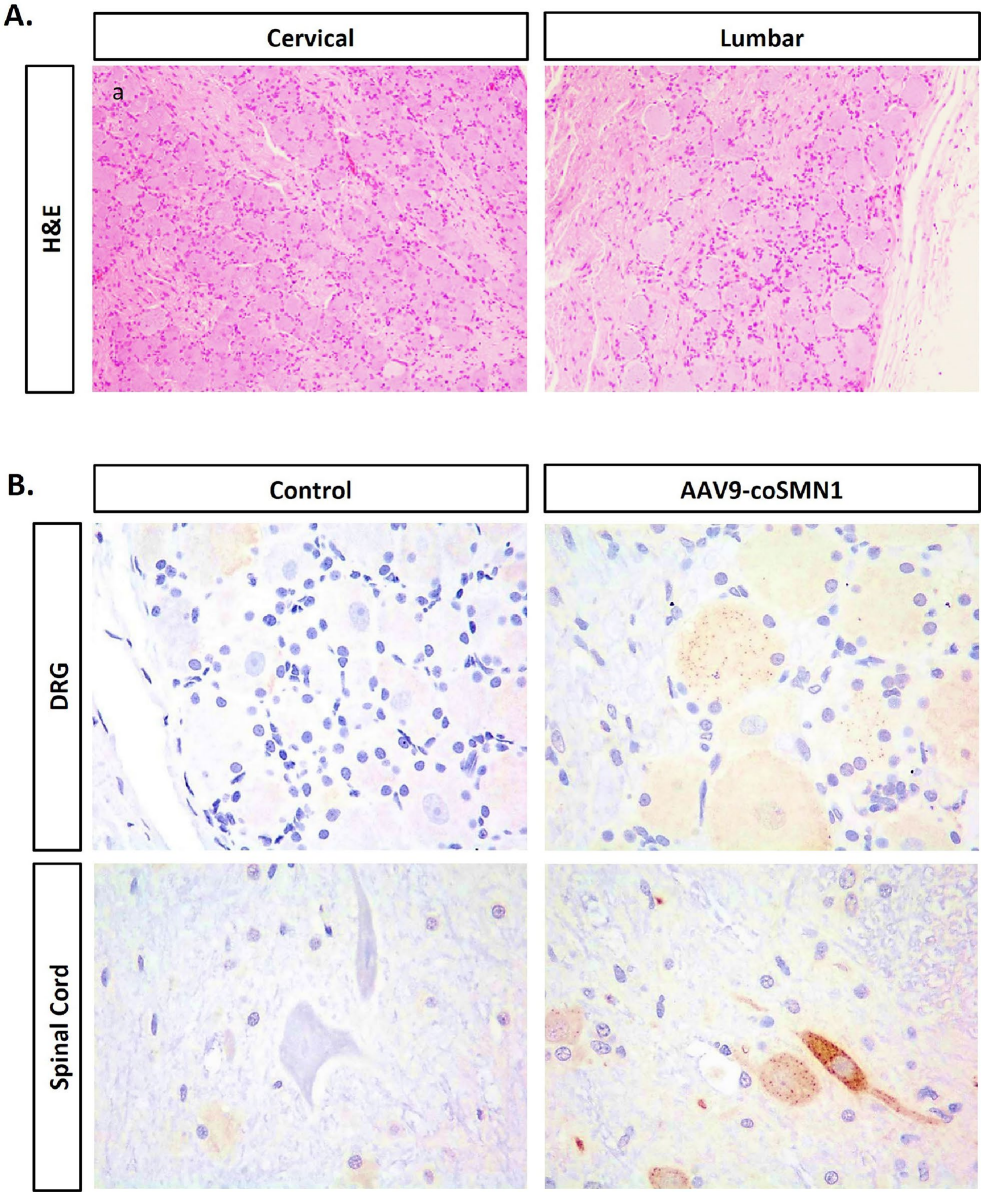
Histological Analysis of DRG & Spinal cord Following AAV9-coSMN1 treatment in Non-Human Primates. **A** H&E staining of DRG day 91 post-injection for analysis, with no significant pathological changes observed post-treatment. **B** SMN immunohistochemistry in DRG and spinal cord demonstrating an upregulation of SMN expression in the treated groups. Representative pictures showing minor detectable sign of toxicity. The sample size of the treated is 8 (male n = 4 and female n = 4) and the control is 4 (male n = 2 and female n = 2)

Histological examination using hematoxylin and eosin (H&E) staining revealed no signs of inflammatory cell infiltration or neuronal necrosis in the DRG and spinal cord of AAV9-coSMN1-treated monkeys (Fig. 7A). To further confirm the expression of SMN protein and assess any potential adverse effects, immunohistochemical staining was performed. The analysis showed successful and robust SMN expression in the neurons of the treated group, with no evidence of inflammation or other pathological changes (Fig. 7B).

These findings suggest that intrathecal administration of AAV9-coSMN1 is well-tolerated in the DRG and spinal cord of cynomolgus monkeys, supporting its favorable safety profile in the central nervous system.

## Discussion

Gene therapy has emerged as a highly effective and promising approach for treating spinal muscular atrophy, particularly through the delivery of SMN protein to motor neurons, which restores muscle function and prolongs survival (Lin C et al. 2024; Wang J H et al. 2024). AAV-mediated SMN delivery has been especially successful in SMA Type 1 model mice, significantly extending their lifespan and improving disease outcome (Meyer K et al., 2014). Clinical trials involving intravenous AAV9-SMN1 have also shown positive results, though concerns remain regarding side effects such as elevated liver enzymes, especially in older patients (NCT04851873). Moreover, the efficiency of AAV9-mediated gene transduction in spinal motor neurons diminishes with age, necessitating the exploration of alternative delivery administration methods that could be more broadly applicable across different SMA phenotypes.

A serial mice models including SMNΔ7, Taiwanese model, *Smn2B/−*, type II/III Burgheron, hemi-hybrid, Burghes’severe, SMNRT, moderate type II (*Smn*^+/−^，*SMN*2, *SMN*Δ7) and neuronal Smn deletion (NSE-Cre^+^ SmnF7/F7) have been used in therapeutic intervention studies (Ellie M. Chilcott et al. 2022). The SMA∆7 mouse model has shown that intracerebroventricular injection can effectively treat Type 1 SMA, significantly extending survival with a lower therapeutic dose (Kathrin M et al. 2015). These results suggest that cerebrospinal fluid (CSF)-mediated gene delivery could be advantageous for severe SMA phenotypes. Furthermore, greater survival benefits when genetic therapy was given to Taiwanese mice than in treated SMNΔ7 mice. Building on this approach, our study investigates whether AAV9-coSMN1 can achieve long-term efficacy in the milder Type 3 SMA mice model. Our findings indicate that AAV9-coSMN1 effectively rescues motor neurons and preserves tail integrity in Type 3 SMA mice, supporting its potential as a treatment for this SMA subtype.

To enhance the therapeutic potential of SMN protein, we implemented codon optimization, increasing SMN1 gene expression efficiency by aligning it with human codon preferences. While species differences may limit the full reflection of these advantages in SMA model mice, in vitro experiments confirmed that coSMN1 significantly outperforms the native SMN1 codon sequence in terms of expression efficiency, leading to higher SMN protein levels. These findings are expected to be crucial for the success of future clinical trials.

Skeletal muscle atrophy, alongside spinal motor neuron degeneration, is a significant aspect of SMA pathology (Aasdev A et al. 2024). While SMA is primarily neurogenic, SMN deficiency also leads to intron retention abnormalities and mitochondrial dysfunction in muscle tissue (Mohini J et al. 2017). In type 3 SMA mice, this dysfunction manifests as spinal motor neuron loss in the anterior horn and gradual tail necrosis (HM Hsieh-Li et al. 2000). Additionally, mitochondrial dysfunction and compensatory hypertrophy were observed in the skeletal muscles of these mice, indicating that, despite milder motor symptoms, Type 3 SMA mice still display significant molecular pathology in muscle tissue. Myogenesis and mitochondrial damage in the SMA population have been confirmed through research (Ripolone M et al. 2015). Notably, a single injection of AAV9-coSMN1 significantly improved muscle fiber area in the gluteus maximus and quadriceps femoris, restoring mitochondrial function and alleviating hypertrophy. Thus, AAV9-coSMN1 not only rescues motor neurons but also positively influences muscle health.

Biodistribution studies in both mice and cynomolgus monkeys demonstrate that a single intracerebroventricular or intrathecal injection of AAV9-coSMN1 results in widespread distribution across the central nervous system, significantly increasing SMN protein levels. This highlights the efficiency of CSF-mediated AAV infusion for gene delivery to the CNS. Furthermore, this approach could be extended to other neurological disorders, such as Dravet syndrome and X-linked adrenoleukodystrophy (X-ALD), offering broad therapeutic potential.

CSF-mediated AAV injection not only efficiently delivers the target gene to the CNS but also enables AAV9 to traverse the blood–brain barrier, facilitating gene delivery to peripheral organs (Kathrin M et al. 2015). In our study, detectable levels of the vector gene were found in the heart, liver, kidneys, and muscles of cynomolgus monkeys, with concentrations ranging from 10E + 5 to 10E + 7 copies/μg of genomic DNA. This biodistribution pattern supports the versatility of this approach for treating various SMA phenotypes and potentially other systemic diseases.

Inflammation of DRG is a known risk in AAV gene therapy, often triggered by excessive gene delivery and immune responses (Hordeaux J et al. 2018; Hordeaux J, et al. 2020). However, in this study, at a dose of 4.67E + 13 vg/animal, no toxic inflammatory responses were observed in the DRG, despite robust SMN protein expression. This indicates that AAV9-coSMN1 has a favorable safety profile, even at higher expression levels.

Notably, the delivery volume for ICV. or IT. Injection is less than 2 ml, therefore, a high quality and concentration of vector is required. AAV production often relies on systems like the triple plasmid co-transfection or the Sf9-baculovirus system. It is imperative to recognize that the production quality serves as a paramount determinant for ensuring safety (Liu S et al. 2024). Future research could explore how these production methods impact the efficacy and safety of AAV9-coSMN1, particularly in clinical settings.

The primary toxic side effects of AAV gene therapy are immune responses against the AAV capsid or other components, leading to complications such as elevated aminotransferase levels, myocarditis, and DRG inflammation. Notably, AAV vectors produced using the HEK293 system may exacerbate these immune reactions due to higher residual host genome content in the capsid, highlighting the need for further research and optimization.

In conclusion, our preclinical efficacy study of AAV9-coSMN1 in a Type 3 SMA mouse model demonstrated long-term motor neuron rescue, significant tail preservation, and improvements in skeletal muscle histopathology. Additionally, under GLP conditions, we confirmed the safety, biodistribution, pharmacology, and toxicology of AAV9-coSMN1 in both mice and cynomolgus monkeys. These findings provide critical data supporting the clinical application of AAV9-coSMN1 and highlight its potential as a safe and effective treatment for SMA.

## Supplementary Information

Additional file 1.

Additional file 2.

Additional file 3.

## Data Availability

No datasets were generated or analysed during the current study.

## Abbreviations

SMA: Spinal muscular atrophy
AAV9: Adeno-associated virus serotype 9
coSMN1: Codon-optimized SMN1
SMN: Survival motor neuron
qPCR: Quantitative Polymerase Chain Reaction
CNS: Central nervous system
CAI: Codon adaptation index
ChAT: Choline acetyltransferase
BHK21: Baby hamster kidney 21 cells
HEK293: Human embryonic kidney 293 cells
MOI: Multiplicity of infection
ICV: Intracerebroventricular
PND: Postnatal day
SDH: Succinate dehydrogenase
GLP: Good Laboratory Practice
DRG: Dorsal root ganglia
H&E: Hematoxylin and eosin
CSF: Cerebrospinal fluid
X-ALD: X-linked adrenoleukodystrophy
