# Supplementary material for: Preclinical evaluation of AAV9-coSMN1 gene therapy for spinal muscular atrophy: efficacy and safety in mouse models and non-human primates
Source: Mol Med. 2025 Apr 29;31:158. doi: 10.1186/s10020-025-01207-4 (PMC12042585; doi:10.1186/s10020-025-01207-4)
Supplement: Supplementary file 3 — Additional file 3. [file 10020_2025_1207_MOESM3_ESM.pdf]

Supplement 3. hSMN1 and coSMN1 sequences

hSMN1

>>ATGGCGATGAGCAGCGGCGGCAGTGGTGGCGGCGTCCCGGAGCAGGAGGATTCCGTG
CTGTTCCGGCGCGGCACAGGCCAGAGCGATGATTCTGACATTTGGGATGATACAGCACTG
ATAAAAGCATATGATAAAGCTGTGGCTTCATTTAAGCATGCTCTAAAGAATGGTGACATTTG
TGAAACTTCGGGTAAACCAAAAACCACACCTAAAAGAAAACCTGCTAAGAAGAATAAAAG
CCAAAAGAAGAATACTGCAGCTTCCTTACAACAGTGGAAAGTTGGGGACAAATGTTCTGCC
ATTTGGTCAGAAGACGGTTGCATTTACCCAGCTACCATTGCTTCAATTGATTTTAAGAGAGA
AACCTGTGTTGTGGTTTACACTGGATATGGAAATAGAGAGGAGCAAAATCTGTCCGATCTA
CTTTCCCCAATCTGTGAAGTAGCTAATAATATAGAACAGAATGCTCAAGAGAATGAAAATG
AAAGCCAAGTTTCAACAGATGAAAGTGAGAACTCCAGGTCTCCTGGAAATAAATCAGATAA
CATCAAGCCCAAATCTGCTCCATGGAACTCTTTTCTCCCTCCACCACCCCCCATGCCAGGG
CCAAGACTGGGACCAGGAAAGCCAGGTCTAAAATTCAATGGCCCACCACCGCCACCGCCA
CCACCACCACCCCACTTACTATCATGCTGGCTGCCTCCATTTCCTTCTGGACCACCAATAAT
TCCCCCACCACCTCCCATATGTCCAGATTCTCTTGATGATGCTGATGCTTTGGGAAGTATGT
TAATTTCATGGTACATGAGTGGCTATCATACTGGCTATTATATGGGTTTCAGACAAAATCAA
AAAGAAGGAAGGTGCTCACATTCCTTAAATTAA*

coSMN1

>>ATGGCCATGAGCTCCGGAGGATCTGGAGGAGGCGTGCCTGAGCAGGAGGACAGCGTG
CTGTTCCGGAGAGGCACCGGCCAGAGCGACGATTCCGACATCTGGGACGATACAGCCCTG
ATCAAGGCCTACGATAAGGCCGTGGCCTCCTTTAAGCACGCCCTGAAGAACGGCGATATCT
GCGAGACCAGCGGCAAGCCTAAGACCACACCAAAGCGGAAGCCCGCCAAGAAGAACAA
GTCCCAGAAGAAGAATACAGCCGCCTCTCTGCAGCAGTGGAAAGTGGGCGACAAGTGCTC
CGCCATCTGGTCTGAGGATGGCTGTATCTATCCCGCCACCATCGCCTCCATCGACTTCAAG
CGGGAGACCTGCGTGGTGGTGTACACAGGCTATGGCAACAGAGAGGAGCAGAATCTGAG
CGATCTGCTGTCCCCAATCTGTGAGGTGGCCAACAATATCGAGCAGAACGCCCAGGAGAA
CGAGAATGAGTCTCAGGTGAGCACAGACGAGTCCGAGAACAGCCGGAGCCCAGGAAACA
AGTCTGATAATATCAAGCCTAAGTCTGCCCCATGGAACAGCTTCCTGCCCCCTCCACCCCCT
ATGCCAGGACCTAGGCTGGGACCAGGCAAGCCCGGCCTGAAGTTTAATGGACCTCCCCCA
CCTCCTCCACCACCACCTCCACACCTGCTGAGCTGCTGGCTGCCACCTTTCCCATCCGGAC
CACCAATCATCCCTCCACCACCTCCAATCTGTCCTGACAGCCTGGACGATGCCGATGCCCT
GGGCTCTATGCTGATCAGCTGGTACATGTCCGGCTATCACACCGGCTACTATATGGGCTTTA
GGCAGAACCAGAAGGAGGGCCGCTGTTCCCACTCTCTGAATTGA
